# Genomic profiling of exogenous abscisic acid-responsive microRNAs in tomato (*Solanum lycopersicum*)

**DOI:** 10.1186/s12864-016-2591-8

**Published:** 2016-06-03

**Authors:** Hai-Yang Cheng, Yan Wang, Xiang Tao, Yan-Fen Fan, Ya Dai, Hong Yang, Xin-Rong Ma

**Affiliations:** Chengdu Institute of Biology, Chinese Academy of Sciences, No 9, Section 4, Renmin South Road, Chengdu, 610041 China; University of Chinese Academy of Sciences, Beijing, 100049 China

**Keywords:** microRNAs, Abscisic acid (ABA), Tomato (*Solanum lycopersicum*), RNA-Seq, Transcription factor, Condition stress tolerance, Pathogen resistance

## Abstract

**Background:**

Plant microRNAs (miRNAs) are involved in various biological pathways and stress responses as negative regulators at the posttranscriptional level. Abscisic acid (ABA) is a key signaling molecule that mediates plant stress response by activating many stress-related genes. Although some miRNAs in plants are previously identified to respond to ABA, a comprehensive profile of ABA-responsive miRNAs has not yet been elucidated.

**Results:**

Here, we identified miRNAs responding to exogenous application of ABA, and their predicted target genes in the model plant organism tomato (*Solanum lycopersicum*). Deep sequencing of small RNAs from ABA-treated and untreated tomatoes revealed that miRNAs can be up- or down-regulated upon treatment with ABA. A total of 1067 miRNAs were detected (including 365 known and 702 candidate novel miRNAs), of those, 416 miRNAs which had an abundance over two TPM (transcripts per million) were selected for differential expression analysis. We identified 269 (180 known and 89 novel) miRNAs that respond to exogenous ABA treatment with a change in expression level of |log_2_FC|≥0.25. 136 of these miRNAs (90 known and 46 novel) were expressed at significantly different levels |log_2_FC|≥1 between treatments. Furthermore, stem-loop RT-PCR was applied to validate the RNA-seq data. Target prediction and analysis of the corresponding ABA-responsive transcriptome data uncovered that differentially expressed miRNAs are involved in condition stress and pathogen resistance, growth and development. Among them, approximately 90 miRNAs were predicted to target transcription factors and pathogen resistance genes. Some miRNAs had functional overlap in biotic and abiotic stress. Most of these miRNAs were down-regulated following exposure to exogenous ABA, while their related target genes were inversely up-regulated, which is consistent with their negative regulatory role in gene expression.

**Conclusions:**

Exogenous ABA application influences the composition and expression level of tomato miRNAs. ABA mainly down-regulates miRNAs that their target genes involve in abiotic stress adaption and disease resistance. ABA might increase expression of stress-related genes via miRNA-mediated posttranscriptional regulation, and our results indicate that ABA treatment has the potential to improve both abiotic stress tolerance and pathogen resistance. This study presents a comprehensive profile of ABA-regulated miRNAs in the tomato, and provides a robust database for further investigation of ABA regulatory mechanisms.

**Electronic supplementary material:**

The online version of this article (doi:10.1186/s12864-016-2591-8) contains supplementary material, which is available to authorized users.

## Background

MicroRNAs (miRNAs) are endogenous non-coding RNAs around 22 nucleotides in length. Since the initial discovery of miRNAs as essential regulators of development in the nematode *Caenorhabditis elegans*, thousands of miRNAs have been identified in animal and plant genomes [1–3]. Mature miRNAs are derived from single-stranded RNA transcripts that contain an imperfect stem-loop secondary structure, which forms a hairpin structure that is processed by Dicer-like 1 into the miRNA duplex in the nucleus, and are then transported to the cytoplasm in plants [4]. miRNAs can complement corresponding target mRNAs to induce RNA interference, thereby inhibiting mRNA translation [5]. Plant miRNAs are involved in a range of activities including responses to adverse environments such as drought, temperature, salt, nutrient starvation, and heavy metal stresses [3, 6], as well as defense responses against pathogen infections [7].

Tomato (*Solanum lycopersicum*) is one of the most important fruit vegetable crops cultivated worldwide, and is also a model organism in plant scientific research. Following the sequencing of its complete genome, it is becoming possible to predict, characterize, and validate miRNAs from the tomato (sly-miRNAs) [8, 9], yet only ~50 mature tomato miRNAs have been reported in the miRBase database to date (http://www.mirbase.org/). Some of these miRNAs are well characterized; for example, miR395, miR398, and miR399 are linked to nutrient deficiency stress, and miR399 is specifically induced in response to phosphate starvation [10–12]. Recently, some putative novel miRNAs in small RNA (sRNA) libraries of tomato fruit and leaf tissue were discovered by cloning and sequencing [13]. Using computational approaches, Yin et al. [14] detected 21 conserved miRNAs and their 57 potential target genes, while Kim et al. [15] identified 12 conserved miRNAs and predicted 417 potential target genes. However, a large number of miRNAs remain to be discovered, and the functions of most miRNAs remain unknown.

Abscisic acid (ABA) is a major phytohormone that regulates a broad range of plant traits and physiological processes. As a central regulator of stress response in plants, ABA triggers major changes in gene expression and physiological responses to adapt to environmental conditions, as well as in the regulation of plant immune responses that protect the plants against pathogens [16–18]. During these stress-response processes, miRNA expression levels also change in response to ABA to regulate gene expression. The vital roles of some miRNAs in the ABA response have been identified in several plants [19]. For instance, during seed germination of *Arabidopsis thaliana*, miR159 accumulates and regulates Myb proto-oncogene 33 (*MYB33*) and *MYB101* transcription factor expression levels under exogenous ABA application and water deficit treatments [20, 21]. Additionally, miR169 targets the nuclear transcription factor Y (*NFYA*), which plays an important role in drought responses. Exposure to ABA or abiotic stresses greatly induces *A. thaliana NFYA5* transcripts but leads to decreased levels of miR169 [21, 22]. Moreover, miR167 and miR143 respond to exogenous ABA, and regulate abiotic stress adaptation in *Oryza sativa* [23]. These studies demonstrate that some miRNAs are involved in the ABA response and stress adaptation. In addition, some miRNAs such as miR160, miR167, miRNA172, miR158, miR159, miR165/166, miR319, and miR393 are involved in pathogen defense [5, 7]. However, it is unclear if miRNAs associated with pathogen defense also respond to ABA.

Small RNA (sRNA) digitalization analysis based on Illumina high-throughput sequencing takes the advantages of a small sample requirement, high throughput and accuracy, and simply operated automatic platform [2, 24, 25]. This allows for the comprehensive identification of sRNAs of particular species under certain conditions, predicting novel miRNAs, and constructing sRNA differential expression profiles between samples. Therefore, it is now possible to identify novel and low abundance miRNAs using high-throughput sequencing technologies targeting entire genomes [26].

In this study, we carried out comprehensive profiling of tomato leaf miRNAs and analyzed them using high-throughput sequencing. In our previous study, we assembled the transcriptome of tomato (*Solanum lycopersicum*) in response to exogenous application of ABA, and we identified many genes related to pathogen resistance and stress tolerance in response to ABA, including transcription factors and pathogen resistant genes [27]. Here, we identified a number of known and novel miRNAs that respond to ABA, and predicted their potential targets. Moreover, combined with previous comparative transcriptome datasets, we analyzed the expression levels between miRNAs and their target genes and discussed the potential miRNA functions with respect to tomato biotic and abiotic tolerances. This study presents the global expression analysis of ABA-regulated miRNAs in tomato, which enriches the tomato miRNA database and provides a prospective for investigating miRNA function in response to ABA, environmental stress, and pathogen resistance.

## Results and discussion

### Global miRNA profile analysis

We constructed sRNA libraries from tomato leaves sprayed with water (control, C1D) or ABA solution (ABA treatment, A1D) using the HiSeq 2000 system. After we filtered the low quality and contaminated reads, a total of 18,495,769 and 17,947,473 clean reads (14–30 nt) were generated from the C1D and A1D libraries, respectively. Upon removing the adaptor sequences, polyA-containing sequences, and sequences shorter than 14 nt, we obtained 18,179,025 genome-matched reads in control (C1D) and 17,763,348 genome-matched reads in ABA-treated tomato (A1D). These represent 5,345,714 and 5,601,375 unique sRNA sequences for the control and ABA treatment respectively, suggesting that the ABA treatment induced the biogenesis of a high number of unique sRNAs (Table 1). We then categorized and annotated RNA reads into miRNAs, transfer (t)RNAs, small interfering (si)RNAs, small nucleolar (sno)RNAs, ribosomal (r)RNAs, repeat regions, and exon and intron RNA based on genomic location and function analysis.

**Table 1:** The distribution of various small RNA types in control and ABA-treated tomato plants

Category	Control				ABA treatment			
	Unique	Percent	Total reads	Percent	Unique	Percent	Total reads	Percent
Total	5345714	100.00 %	18179025	100.00 %	5601375	100.00 %	17763348	100.00 %
Exon antisense	87855	1.64 %	312186	1.72 %	90115	1.61 %	317073	1.78 %
Exon sense	166193	3.11 %	499371	2.75 %	156483	2.79 %	493025	2.78 %
Intron antisense	301541	5.64 %	854012	4.70 %	319216	5.70 %	932614	5.25 %
Intron sense	385395	7.21 %	1307703	7.19 %	404425	7.22 %	1418817	7.99 %
miRNA tags	39329	0.74 %	1985940	10.92 %	35072	0.63 %	1732993	9.76 %
rRNA	89865	1.68 %	1524613	8.39 %	83003	1.48 %	1175035	6.61 %
repeat	817623	15.29 %	2663412	14.65 %	874556	15.61 %	2942990	16.57 %
snRNA	3073	0.06 %	7334	0.04 %	3106	0.06 %	7605	0.04 %
snoRNA	1098	0.02 %	1914	0.01 %	1187	0.02 %	2206	0.01 %
tRNA	7638	0.14 %	284344	1.56 %	8251	0.15 %	246732	1.39 %
Un-annotated	3446104	64.46 %	8738196	48.07 %	3625961	64.73 %	8494258	47.82 %

**Fig. 1:**
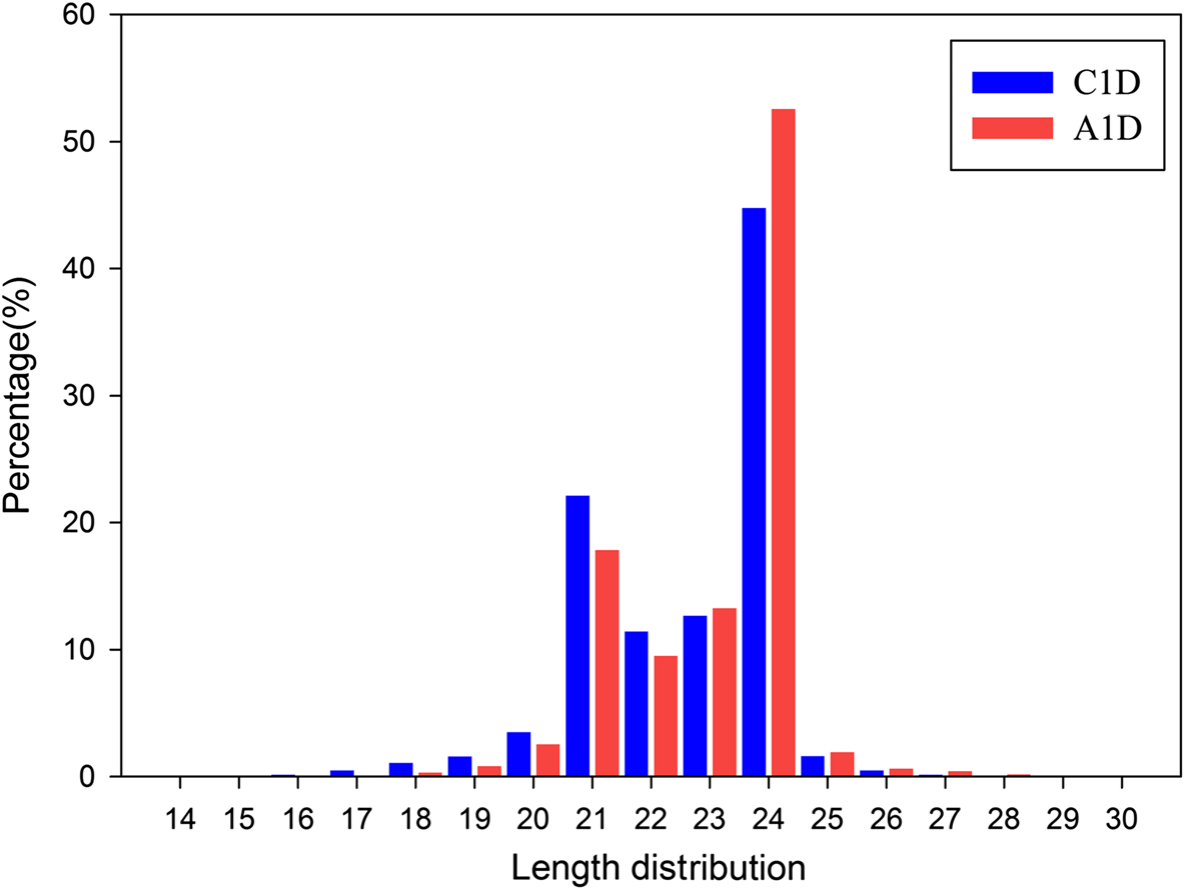
Distribution of small RNAs with different nucleotide lengths in the control (*blue*, C1D) and ABA-treated (*red*, A1D) tomato plants

The length of these sRNA ranged from 14 to 30 nt, with the 21 and 24 nt sRNA classes as the most abundant groups in both libraries, occupying 22.1 and 44.77 % of the total in the control, and 18.8 and 52.54 % of the total in ABA treatment (Fig. 1). This was similar to previous reports in tomato and *Arabidopsis* [28–30]. Furthermore, we analyzed the base bias of miRNA, and found that the first base from the 5′ end has a strong preference of uridine (U) in both control and ABA treatment datasets. This important feature of miRNAs [31, 32] was not altered by the ABA treatment.

We then compared the miRNA profiles between the control and ABA treatment. miRNAs occupied a small portion of the total sRNAs, and their numbers were reduced by ABA treatment, suggesting that ABA may repress miRNA biogenesis (Table 1). The total reads of miRNA fell from 1,985,940 (10.92 %) in C1D to 1,732,993 (9.76 %) in A1D, and the unique miRNA tags reduced from 39,329 (0.74 %) in C1D to 35,072 (0.63 %) in A1D. Furthermore, the 21 nt sRNAs mainly composed of miRNAs, decreased following ABA treatment. These results indicate that ABA diminished miRNA composition and abundance on the whole. Moreover, the abundance of 24 nt sRNAs increased in the A1D compared to C1D, indicating that more 24 nt sRNAs were produced in response to ABA. This was in agreement with the report on heat-responsive miRNAs of *Populus tomentosa* [33]. The 24 nt sRNAs normally belong to siRNA [34], and they may have specific functions in ABA response that requires further investigation. In addition, a total un-annotated reads of 8,494,258 (47.28 %) (3,625,951 unique) in A1D and 8,738,196 (48.07 %) (3,446,104 unique) in C1D were acquired for further predicting novel miRNAs (Table 1).

### miRNA identification and target gene prediction

The previously identified miRNAs are referred to known miRNAs. We searched for known miRNAs in the two sRNA libraries by comparing them with data from tomato and other plant species in miRBase 20.0 (http://www.mirbase.org/). After filtering out known miRNAs, we predicted unidentified miRNAs, referred to as novel miRNAs, using the un-annotated sequence reads in C1D and A1D libraries by Mireap (http://sourceforge.net/projects/mireap). A miRNA precursor has a characteristic stem-loop hairpin secondary structure, which is one of most important feature that separates miRNAs from other endogenous small RNAs [35]. In total, 1067 miRNAs were identified, including 365 known and 702 novel miRNAs (Additional file 1: Table S1, S2 and S3). Additional file 1: Table S3 listed eight novel miRNAs, which were recently named according to updated miRNAase 21.0 database. However, these newly updated miRNA did not affect analysis of the study. The predicted precursor sequences for novel miRNAs preferentially in the secondary structure were listed in additional file (Additional file 1: Table S4). The results indicated that high-throughput RNA-sequencing technology is a powerful technique for large-scale analysis of miRNA expression and identification of novel miRNAs.

Among known miRNAs, five members including sly-miR156, miR167a, miR168a, miR166g-3p, and sly-miR166, showed high abundance with >5000 standardized reads (Transcripts per millions, TPM). In general, ancient miRNAs are more highly conserved and abundant in terrestrial plants [36]. Four miRNAs, sly-miR4376, miR172a, sly-miR6027 and miR7822, exhibited level with 1000–5000 TPM. Furthermore, 61 known miRNAs were expressed at 100–1000 TPM, 136 miRNAs at 10–100, and 92 miRNAs at 2–10, leaving 67 miRNAs expressed below two TPM.

As compared with known miRNAs, the novel miRNAs displayed lower expression levels (Additional file 1: Table S5 and S6). Only four (novel_mir_440, novel_mir_421, novel_mir_441, and novel_mir_253) had high abundance at 1000–3000 TPM. In addition, we also identified six novel miRNAs with expression level at 100–1000 TPM, 32 miRNAs at 10–100, and 76 miRNAs at 2–10. The results suggested that newly identified miRNAs were generally expressed at lower levels, which is in agreement with the previous report in *Arabidopsis thaliana* [32]. Overall, the finding of novel miRNAs in tomato has provided enriched insight into the plant miRNA dataset. However, the functions of these novel miRNAs need to be further demonstrated.

Target prediction of miRNA led to the identification of the genes regulated by those miRNAs. In our analysis, we identified 170 known and 237 novel miRNAs with predicted target genes. Most of those miRNAs were shown to have multiple target genes, which are consistent with other reports [37]. For instance, miR6024-3p, miR5658, miR5139, sly-miR156, novel_mir_156, and novel_mir_447 had 71, 45, 15, 11, 6 and 2 predicted target genes, respectively. The results indicated that the single miRNA might participate in multiple signal pathways and possess wide-ranging functions in tomato. Overall, the number of predicted target genes of known miRNAs was much greater than that of novel miRNAs. Reportedly, conserved miRNAs contained more target genes [37, 38]. Our results also support the previous finding that evolutionarily conserved miRNAs exert more functions in vivo [37].

### Exogenous ABA regulates tomato miRNA expression

**Fig. 2:**
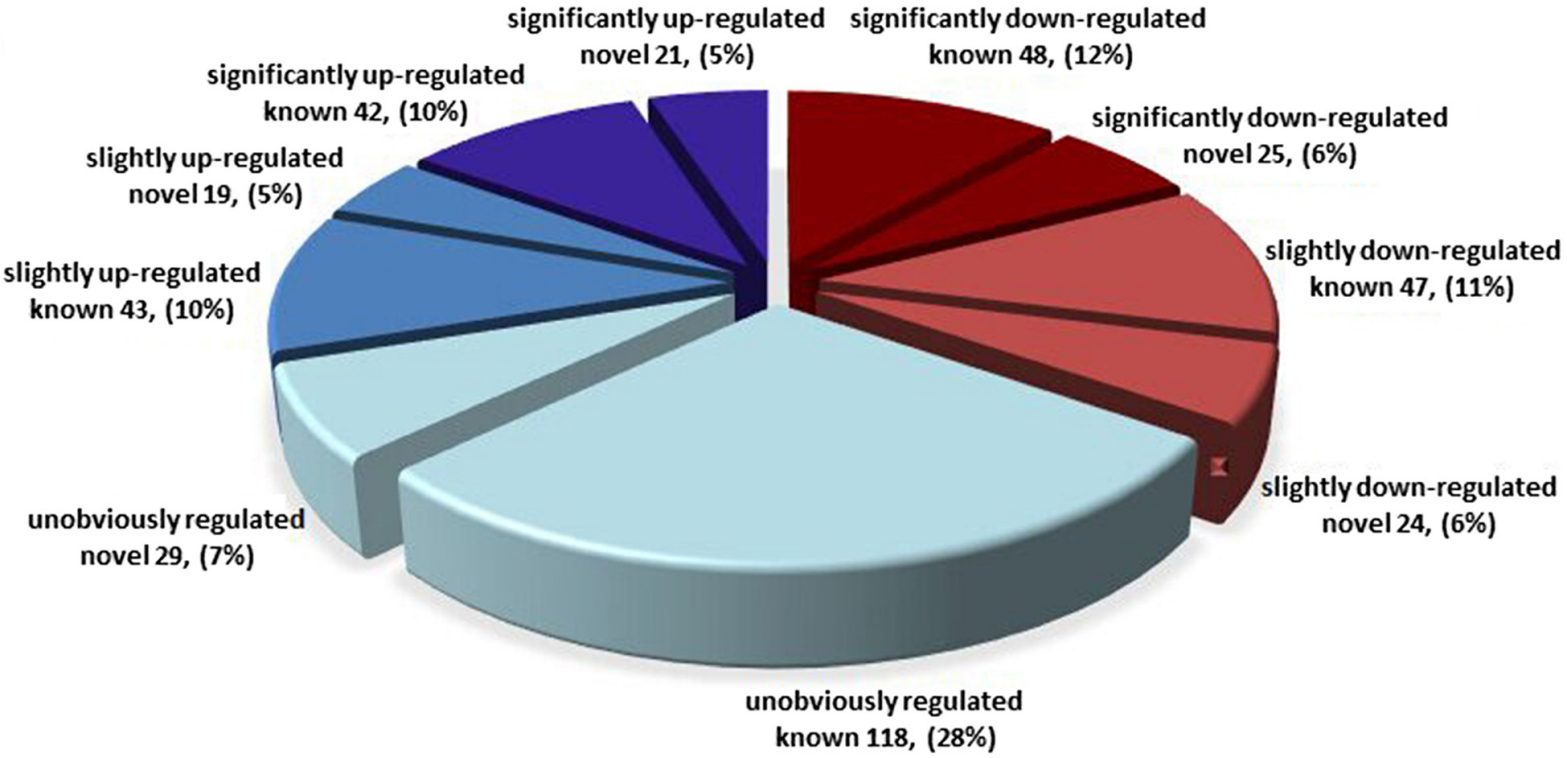
Differential expression analysis of miRNAs between the control (C1D) and ABA-treatment (A1D) libraries. The miRNAs with expression level ≥2 TPM were listed (416 miRNAs). miRNAs that satisfied the criteria “|log_2_FC| ≥1” and *P* <0.05 were considered as “significantly up-regulated” or “significantly down-regulated”. miRNAs that differed by 1>|log_2_FC|≥0.25 were assigned to “slightly up-regulated” or “slightly down-regulated”. miRNAs that did not fall into either of the above-mentioned categories were characterized as “unobviously regulated”

To evaluate the regulatory roles of ABA on miRNA expressions, we compared the differential expression profiles of miRNAs between the control and ABA-treatment. To minimize noise and improve accuracy, the miRNAs that had TPM value less than two were removed, leaving 416 miRNAs for further differential expression analysis. Changes in the expression level of at least |log_2_fold-change (log_2_FC)| ≥0.25 were recognized as a response to ABA treatment (Fig. 2, Additional file 1: Table S5 and S6). Accordingly, a total of 269 miRNAs exhibited differential expression in response to exogenous application of ABA, including 136 (73 down and 63 up) strongly altered miRNAs with |log_2_FC|≥1 and *P* <0.05, and 133 (71 down and 62 up) slightly altered with 1>|log_2_FC|≥0.25. In addition, 147 miRNAs did not displayed change in expression levels with |log_2_FC|<0.25.

**Fig. 3:**
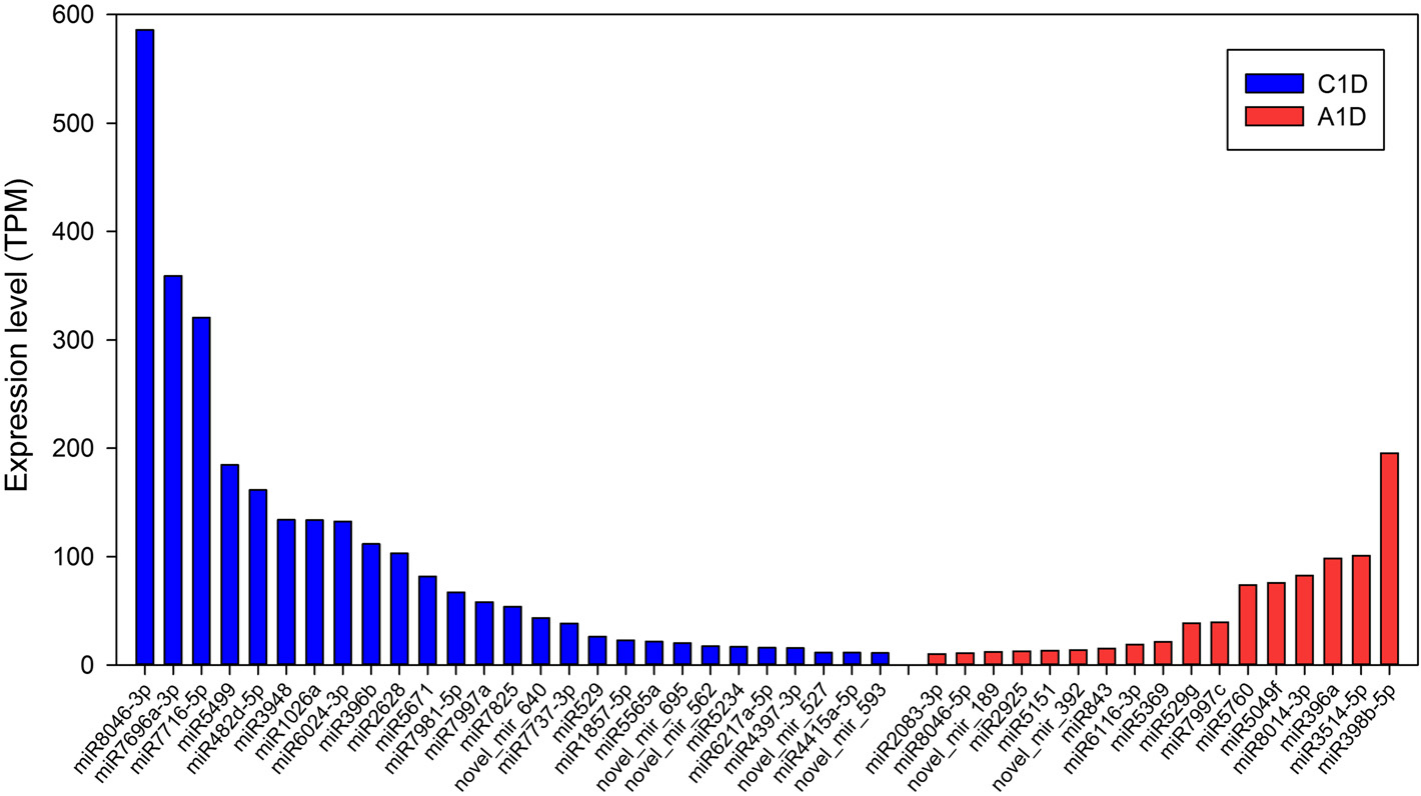
The miRNAs completely repressed or induced by ABA treatment. Graph contains miRNAs that were completely repressed or induced upon ABA-treatment, and had a minimum TPM of ten. *Blue* denotes miRNAs expressed in control plants, and *red* denotes miRNAs expressed in the ABA-treated plants

**Fig. 4:**
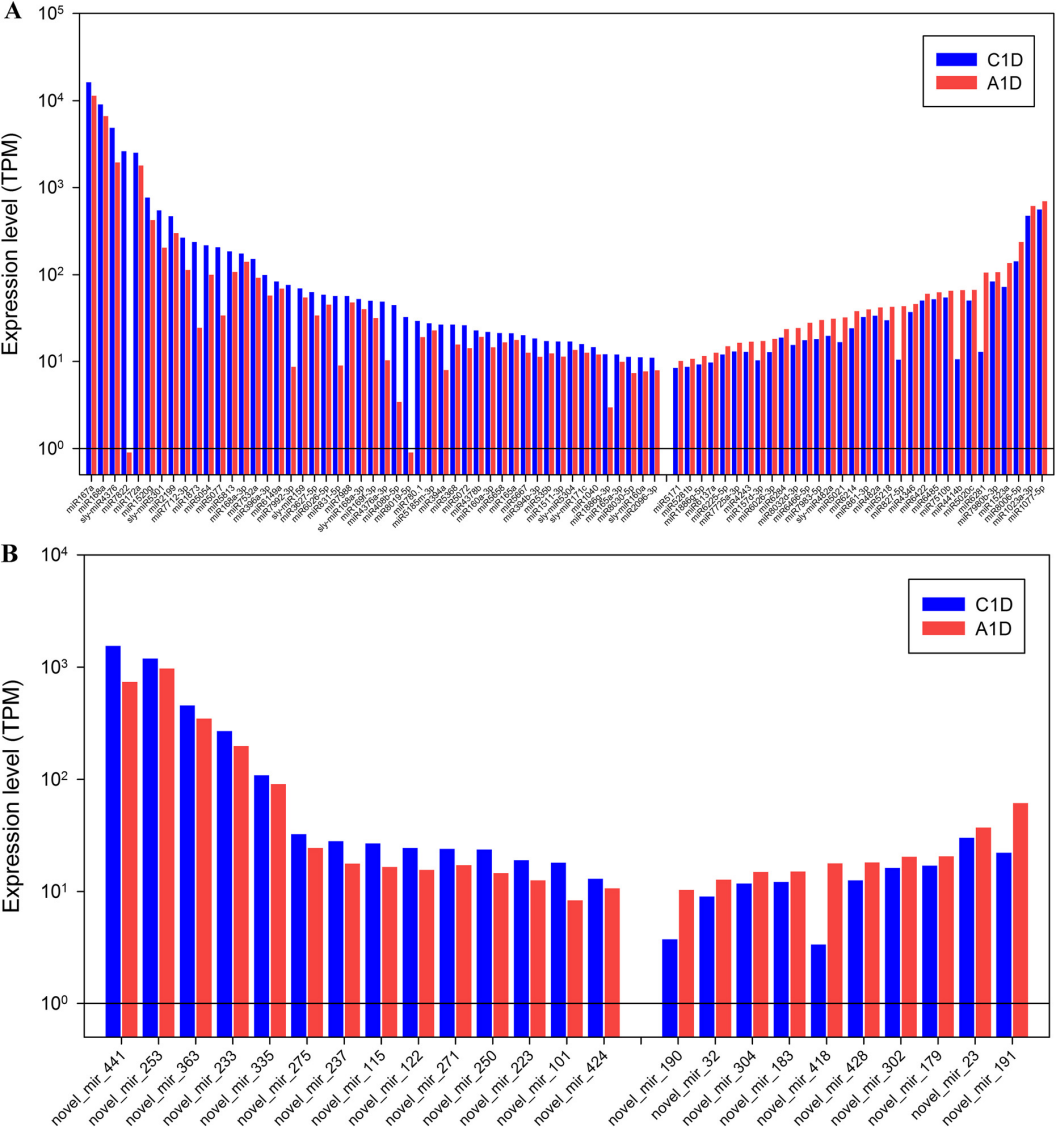
Comparison of expression levels in miRNAs that were shared between the control (C1D, *blue*) and ABA (A1D, *red*) treated tomato plants. The miRNAs that differed by |log_2_FC| ≥0.25 were shown. Graph contains (**a**) the differentially expressed known miRNAs, and (**b**) novel miRNAs. For both panels, miRNAs that had a minimum TPM value of ten in one library are listed

After removing the miRNAs with |log_2_FC| <0.25, 269 miRNAs (180 known and 89 novel) were left for further expression analysis. Among them, 32 known and 23 novel candidates were only detected in the control, while 36 known and 17 novel ones were seen only in ABA-treatment, indicating that ABA completely inhibited or induced expression of these miRNAs in ABA-treatment. Figure 3 shows the 27 completely inhibited and 17 induced miRNAs with over ten TPM. Among the other miRNAs shared between the two libraries, 112 known and 49 novel miRNAs exhibited differential expressions. The shared miRNAs with over ten TPM presented 90 down-regulated miRNAs and 61 up-regulated miRNAs, as shown in Fig. 4a and b. In these highly expressed miRNAs, not only the number but also the expression abundance of the down-regulated miRNAs were much more than that of the up-regulated. We analyzed the corresponding transcriptome data and identified that the expression level of the miRNA target genes were generally increased upon treatment with ABA (Additional file 1: Table S7), indicating miRNAs negatively regulated target gene expressions. For example, miR6024-3p, showed a fall in expressed counts from 132.63 to 0.01 TPM following the ABA treatment, and was predicted to act on 71 genes corresponding to 95 transcripts in our ABA-responsive transcriptome. The expressions of these transcripts exhibited that 51 members were elevated, ten were reduced and 36 were unaltered [27]. The results demonstrate that miRNAs responded to exogenous application of ABA, and induced changes in gene expression regulation.

We also conducted a function analysis of target genes of the miRNAs, and found that the differentially expressed miRNAs were involved in condition stress and pathogen resistance, growth and development. Here, we highlight those miRNAs that target transcription factors and defense related genes (Additional file 1: Table S8).

### The miRNAs that target transcription factors

Transcription factors (TFs) interact with cis-elements of responsive gene promoter regions, controlling the expression of many downstream genes, and triggering cascade reactions of many physiological processes and biochemical reactions in plant cells [39]. A large number of TFs respond to ABA treatment in plant [27, 40]. Here, we identified 31 miRNAs that have one or more predicted targets that are transcription factors. Among them, 12 ABA-responsive miRNAs with differential expression were identified, containing eight up-regulated miRNAs (miR6024-3p, miR7997a, miR172a, miR5658, sly-miR5301, miR169b, sly-miR159 and miR165a-3p), and four down-regulated miRNAs (miR7997c, novel_mir_392, novel_mir_191 and sly-miR171d). These miRNAs are likely to target dozens of genes involving in condition stresses and defense responses, as well as growth and development. Here, our results mainly analyzed the miRNAs which their targets are the transcription factors such as MYB (Myb proto-oncogene protein), AP2/EREBP (apetala 2/ethylene-responsive element-binding factor), bHLH (basic helix-loop-helix), bZIP (basic leucine zipper), NAC (no apical meristem/ATAF/CUP-shaped cotyledons), and MADS-box families (Table 2).

**Table 2:** List of selected miRNAs associated with target genes that are transcription factors and disease resistance proteins

miR ID	C1D (TPM)	A1D (TPM)	Target genes (No.)	Expressed transcript & the number			
				Sum	up-regulated	down-regulated	unchanged
miR6024-3p	132.63	0.01	RLK (STK) (2)	2	2	0	0
			NAC (1)	—			
			NBS-LRR (27)	27	11	1	15
miR482a	33.5	41.77	^a^NBS-LRR (1)	—			
sly-miR482a	3.58	7.43	^a^NBS-LRR (2)	2	1	0	1
sly-miR482c	3.58	7.43	^a^NBS-LRR (6)	4	2	1	1
miR6026-3p	12.82	18.13	^a^NBS-LRR (1)	—			
miR5083	2.15	2.76	NBS-LRR (1)	—			
novel_miR_674	3.47	0.01	NBS-LRR (6)	6	2	1	3
novel_miR_191	22.11	61.36	MYB (2)	2	0	1	1
sly-miR159	69.64	54.49	MYB (5)	10	7	3	0
miR5658	21.23	16.55	MYB (3)	4	2	1	1
			RLK (STK) (1)	—			
			bZIP (1)	3	2	0	1
			Jasmonate ZIM-domain (1)	—	—	—	—
			ERF (2)	—			
miR172a	2508.94	1796.79	AP2/ERFBP (8)	15	5	2	8
miR7997a	58.09	0.01	bZIP (1)	3	2	0	1
miR7997c	0.01	39.58					
miR5813	185.65	107.58	STK (1)	3	2	0	1
miR319b	0.01	2.14	RLK (STK) (1)	—			
miR4376a-3p	48.85	10.3	MAP kinase (STK) (1)	1	1	0	0
novel_miR_418	3.36	17.73	RLK (STK) (1)	2	1	0	1
novel_miR_392	0.01	13.57	type I MADS-box (1)	—			

^a^ These NBS-LRR genes are included in the 27 NBS-LRRs targeted by miR6024-3p

*RLK* receptor-like kinase, *STK* serine/threonine-protein kinase, *NAC* no apical meristem/ATAF/CUP-shaped cotyledons, *NBS-LRR* nucleotide binding site and leucine-rich repeat, *MYB* Myb proto-oncogene protein, *bZIP* basic leucine zipper, *ERF* ethylene-responsive transcription factor, *AP2/EREBP* apetala 2/ethylene-responsive element-binding factor, *MAP* mitogen-activated protein

The MYB family, the largest plant transcription factor family, plays important roles in plant stress tolerance [41, 42]. Target gene prediction showed that three miRNAs with differential expression may target ten *MYB* genes, containing two down-regulated miRNAs (miR5658 and sly-miR159) and one up-regulated miRNA (novel_mir_191). Furthermore, we conducted an analysis of the tomato genome for the target *MYB* genes, and statistical analysis for their corresponding transcripts, and observed that they act on different target gene members and correspond to multiple transcripts. The miR5658 was predicted to target three *MYB* genes corresponding to four transcripts in the ABA-responsive transcriptome dataset, and the analysis of expression indicated that there were two up- and one down- regulated, and one unchanged *MYB* genes in the transcriptome. The sly-miR159 was predicted to target five *MYB*s with 10 transcripts, where seven were up-regulated and three were down-regulated in the transcriptome (Table 2). In addition, the up-regulated novel_mir_191 targets on two *MYB*s, and one decreased and the other had no change [27] (Table 2).

The functions of MYB have been investigated in many plant species such as *Arabidopsis*, maize (*Zea mays*), cotton (*Gossypium hirsutum*), rice (*Oryza sativa*), petunia (*Petunia hybrida*) and apple (*Malus domestica*) [42], and have been shown to act on biotic and abiotic stress, phenylpropanoid metabolism, differentiation, cell shape, formation of B-type cyclin, hormone responses [43–45]. In general, the expressions of these miRNAs were reduced upon ABA-treatment, and the target *MYB* genes elevated when exposed to exogenous ABA (Table 2). Interestingly, up-regulated *MYB* is beneficial to improve plant stress tolerances [42].

We next conducted similar analysis for other miRNAs. Two *bZIP* genes were targeted by miR7997a, miR7997c and miR5658. Interestingly, miR7997a and miR7997c target the same *bZIP*, but their abundance changes were completely opposite. miR7997a was repressed from 58.09 to 0.01 TPM and miR7997c was induced from 0.01 to 39.58 upon ABA treatment. Their target *bZIP* corresponded to three transcripts, with two increased and one no change in the ABA-responsive transcriptome. It probably resulted from the two miRNAs functioning together. In addition, three transcripts, deriving from another *bZIP* gene acted by the down-regulated miR5658, showed that two were increased and one was unaltered. Besides, miR5658 was also predicted to act on a *bHLH*, but did not show any expression difference in our transcriptome dataset. miR165a-3p was predicted to target another *bHLH* whose expression was not detected.

The miR172a and miR5658 were predicted to act on several AP2 (AP2-like ethylene-responsive) transcription factors and two ERF (Ethylene-responsive transcription factor) family genes. These are TFs belong to AP2/EREBP transcription factors family that exert crucial roles not only in adverse stress but also in pathogen resistance. AP2/EREBP can interact with DREs (drought-responsive elements) as a trans-acting factor, triggering downstream gene expression changes to improve stress tolerance in plant [46]. They are also involved in ethylene signaling pathway that is linked to biotic stress responses, protecting plant against pathogen attacks [47]. miR172a, down-regulated from 2508.94 TPM in C1D to 1796.79 in A1D by ABA, was predicted target eight *AP2/EREBP* genes, corresponding to 15 transcripts. Of these transcripts, five showed increased and one showed decreased expression, while the other eight had no obvious change in expression levels. All of them were annotated as pathogenesis-related transcriptional factor, indicating that miR172a plays important roles in pathogen resistance. Additionally, two *ERF1* genes that were targeted by miR5658 were not detected in the transcriptome data.

NAC proteins are a family of plant-specific transcription factors that play a crucial role in plant development and in abiotic and biotic stress responses [48]. Here, one *NAC* gene, no apical meristem (NAM) protein, was targeted by miR6024-3p, and showed no change at transcript level.

The novel_mir_392 was predicted to act on a MADS-box transcription factor one (type I MADS-box). Type I MADS-box proteins are required for plant reproduction, particularly in specifying female gametophyte, embryo, and endosperm development [49, 50]. Although novel_mir_392 showed an elevated expression from 0.01 to 13.57 TPM, we did not detect any change in expression levels in the transcriptome.

*AP2/ERFBP*, *bZIP*, *bHLH*, *MYB* and *NAC* family genes involve in multiple biotic and abiotic stress responses, activating downstream stress responsive genes to improve plant stress resistance [51]. Overall, the expression level of most miRNAs related to above stress-inducible transcription factors were diminished upon exogenous ABA treatment, while their targets were generally up-regulated. In general, the ABA-induced genes were enriched for those encoding proteins involved in stress tolerance [17].

### The miRNAs that target pathogen defense genes

Biotic stresses, including fungal, bacterial and viral pathogens, are a major constraint to crop production [52]. The phytohormone ABA plays multifaceted and crucial roles in plant pathogen resistance. In this study, we identified miRNAs which target nucleotide-binding site and leucine-rich repeat (NBS-LRR), *AP2/EREBP*, serine/threonine-protein kinase (STK), jasmonate ZIM-domain protein genes, and globally analyzed the miRNAs and their target genes. In total, we identified 64 miRNAs involved in regulating the expression of disease resistance genes that respond to exogenous ABA (Additional file 1: Table S8). Among them, 14 were known miRNAs with ≥2 TPM in one sample, and these were selected for target gene analysis. The results showed that most of these miRNAs were down-regulated by ABA (Table 2).

To date, the majority of known plant disease-resistance proteins contain NBS-LRR. NBS-LRR resistance proteins directly or indirectly recognize pathogen avirulence factors, triggering signal transduction cascades that lead to rapid defense responses, hypersensitive reactions, and programmed cell death [53–55]. Here, we predicted that seven miRNAs act on *NBS-LRR* gene members, such as miR6024-3p, miR482a, sly-miR482a, sly-miR482c, and miR5083, miR6026-3p and novel_mir_674.

The miR6024-3p was predicted to target 27 members of *NBS-LRR* resistance genes. Interestingly, these 27 members included all the *NBS-LRR* targets of miR482a (one *NBS-LRR* target), sly-miR482a (2), sly-miR482c (6), and miR6026-3p (1). Analysis of miR6024-3p showed a decrease in expression from 132.63 to 0.01 TPM following ABA treatment, although the abundance of other four miRNAs were elevated from 33.5 to 41.77, 3.58 to 7.43, 3.58 to 7.43 and 12.82 to 18.13 TPM. These four miRNAs were predicted to act together with miR6024-3p, and most of their target *NBS-LRR* showed an increased expression. In the predicted 27 members of *NBS-LRR* resistance genes, 11 transcripts were elevated and one was decreased after ABA treatment, and 15 exhibited less obvious changes (Table 2). A novel miRNA, novel_mir_674 (3.47 to 0.01 TPM), was predicted to target six members of *NBS-LRRs*, where two were elevated, three were unaltered and the other was not detected. In addition, the target of miR5083 was not detected in the transcriptomes.

Globally, the results indicated the miRNA that were more abundant had larger influence on target gene expression. Admittedly, the appearance of the expression levels did not completely conform to the negative correlation between a miRNA and the target gene, but the overall trend was in accordance to this hypothesis. This could be explained by the regulatory complexity of miRNAs, which often have one or more targets, and a gene can also be targeted by multiple miRNAs.

Serine/threonine protein kinase (STK) family genes not only play crucial roles in adaption of abiotic stresses but also in pathogen defense in plant [56]. Here, we predicted four differentially expressed miRNAs that target *STK* genes, including miR5813, miR6024-3p, miR4376a-3p and miR319b (Table 2). A *STK* gene was predicted to be targeted by miR5813, and was down-regulated from 185.65 to 107.58 TPM. And the target gene had three transcripts in the transcriptome, with two that were increased in expression and one remained unchanged. miR4376a-3p was down-regulated from 48.85 to 10.30 TPM under ABA treatment, and was predicted to target seven genes. One of its targets is a mitogen-activated protein (MAP) kinase gene, which belongs to STK family. We compared the related transcriptome data and found one transcript that showed increased expression. MAP kinase is involved in the sphingolipid elicitor-dependent defense signaling pathway, which acts downstream of the heterotrimeric G protein alpha subunit and small GTPase RAC1, and may regulate the expression of various genes involved in biotic and abiotic stress responses [57].

Moreover, the receptor-like kinase (RLK), which also belongs to STK family, is implicated in plant pathogen interaction and defense responses [58]. The targets of miR6024-3p comprise two *RLK* genes, and both showed an increase in expression in the transcriptome upon ABA treatment. However, the novel miRNA novel_mir_418 was up-regulated (from 3.36 to 17.73 TPM) upon ABA treatment, and was predicted to target *RLK*, which showed one transcript with increased expression and one without change in expression level. In addition, miR319b (from 0.01 to 2.14 TPM) also targets a *RLK* gene, but the target gene transcript was not detected in the transcriptome. Generally, the expressions of the miRNAs described here were reduced by exogenous ABA application, and their targets of STK were elevated (Table 2).

With respect to jasmonic acid signal transduction pathway, a jasmonate ZIM-domain protein 3 encoding gene was identified as a target of miR5658. This protein, a repressor of jasmonate, is negatively regulated by the proteasome in an SCF (COI1) E3 ubiquitin-protein ligase complex-dependent manner [59, 60]. The expression level of miR5658 decreased from 21.23 to 16.55 TPM, but the target transcript was not detected (Table 2).

AP2/EREBP family regulation is usually involved in biotic stress responses, such as pathogen attack and jasmonate and ethylene pathways [61]. Here, we identified two known miRNAs that target *AP2/EREBP*, miR172a and miR5658, which were predicted to target eight and one *AP2/EREBP* genes respectively (Table 2). The expressions of these miRNAs and their targets were analyzed as described above.

Taken together, the miRNAs related to disease resistance showed a tendency to be down-regulated by ABA treatment, and the target disease resistance genes were mainly up-regulated in the transcriptome of ABA treated plants. Reportedly, ABA was considered to have multifaceted role in plant resistance to both biotrophic and necrotrophic fungi and bacteria [62]. Our results suggested that ABA improves broad-spectrum pathogen resistance in tomato, as supported by the years-field trials (data not shown). However, the underlying mechanism of pathogen resistance induced by ABA in plants remains to be fully explored.

A miRNA usually targets multiple genes, which possibly exert roles in different development stages and stresses, playing various functions. Likewise, some genes also are targeted by one or more miRNAs. Accordingly, a miRNA probably is in the presence of functional overlap in biotic and abiotic stress, such as miR6024a-3p, miR172a, miR5658, implying that a complex crosstalk between the global regulation of miRNA metabolism and ABA signaling functions enables the fine-tuning of stress response in plants [63].

### Quantitative real-time-PCR validation of differentially expressed miRNAs from RNA-seq

To confirm the accuracy and reproducibility of our Illumina RNA-seq results, 30 miRNAs were chosen for stem-loop quantitative real-time (qRT) PCR. The primer sequences were designed according to each miRNA sequence (Additional file 1: Table S9). The expressions of selected 30 miRNAs were calculated using q-PCR. Twenty-three out of 30 miRNAs exhibited expression in the same trend with that from the sequence analysis, accounting for 76.67 %, suggesting that RNA-seq data were reliable (Additional file 1: Table S10).

## Conclusions

In the present study, we used genome-wide miRNA profiling to identify multiple miRNAs that responded to exogenous ABA treatment in the tomato. Exogenous ABA application was shown to down-regulate many miRNAs involved in stress tolerance and pathogen resistance, including those targeting genes encoding transcription factors and disease resistance proteins. In general, miRNA expression level changes were negatively correlated with the expression of their target genes. ABA may increase expression of stress-related genes by miRNA-mediated posttranscriptional regulation. Our results indicate that ABA treatment has the potential to improve not only abiotic stress tolerance, but also pathogen resistance in plants by adjusting the expression of miRNAs and their target genes.

## Methods

### Plant materials

The seeds of Tomato cv. Hongtaiyang 903 were purchased from Dalian Tiandi Seed CO, LTD, planted in plastic pots filled with organic loam in April, 2012, and grown in a glasshouse in Chengdu (30.67°N, 104.06°E), Sichuan Province of China. The region had natural photoperiod, irradiance of approximately 150 μmol m^−2^ s^−1^, and the temperature range was 18–25 °C. The seedlings were treated by ABA solution and water as previously described [26]. Briefly, tomato seeds were cultivated in 60 plastic pots, and watered every other day. After germination, four seedlings were placed in each pot. After 45 days, when the plants were at the 5–7 leaf growth stage, the pots were randomly divided into two groups. One group was sprayed with 400 mL of 7.58 μmol L^−1^ ABA solution (treated group), and the other group was sprayed with the same volume of purified water (control group). After 24 h, the young third leaf was picked from ten randomly selected plants from both groups, snap-frozen, and stored in liquid nitrogen [27]. Three or more independent sample pools were collected for each treatment using the same sampling method.

### Small RNA library preparation and sequencing

To identify ABA-responsive miRNAs in the tomato, two sRNA libraries from tomato treated with ABA (A1D) or sprayed with water (C1D, control) were constructed. Total RNA was isolated from C1D and A1D using Trizol reagent (Invitrogen, Carlsbad, CA), following the manufacturer’s protocol. Small RNAs were separated from total RNA by size-fractionation on a 15 % PAGE gel, and RNA molecules in length of 18–30 nt were purified from the gel. After ligated with a pair of adaptors of 5′ (5′-GUUCAGAGUUCUACAGUCCGACGAUC-3′) and 3′ (5′-UGGAAUUCUCGGGUGCCAAGG-3′), small RNA molecules were subjected to reverse transcription-PCR (Superscript II reverse transcriptase, 15 cycles of amplification). The PCR products were used for sequencing by Solexa technology on HiSeq™ 2000 (Illumina, San Diego, CA, USA) by BGI, Shenzhen China.

### Sequence analysis for miRNA identification

The raw data were processed according to Sunkar et al. [64]. After filtering out the low quality reads and trimmed the adaptor sequences, high quality clean sequences of 15–30 nt length were mapped to the tomato genome that was published in 2012 (ftp://ftpmips.helmholtz-muenchen.de/plants/tomato/tomato_enome/ITAG_annotation/ITAG2.3_release/ITAG2.3_cdna.fasta) using the SOAP program [9, 65]. The annotated sequences were further screened to remove rRNA, tRNA, snRNA, snoRNA, and those containing polyA tails by searching against NCBI Genbank database and Rfam database (http://www.sanger.ac.uk/software/Rfam). The remaining sequences were then compared against known plant miRNAs in miRBase (miRBase 20.0) to identify conserved miRNAs. Considering the difference among species, alignments of the cleaned reads to the miRNA precursor/mature miRNA of all plants/animals in miRBase allowed for two mismatches.

Then un-annotated reads were used to predict candidates for novel miRNA using the prediction method developed by Meyers et al. [66]. The software Mireap (http://sourceforge.net/projects/mireap), developed by BGI (Beijing Genomics Institute), was employed to predict novel miRNA candidates. Mireap software predicts novel miRNA by exploring the secondary structure, Dicer cleavage sites, and the minimum free energy of unannotated sRNA tags that can be mapped to the genome [67]. The tags predicted for novel miRNAs according to this method must match the following qualifications: 1) Tags should be unannotated tags that can match the reference genome, aligning to intronic regions or to antisense exon regions; 2) The sequences and structures of the genes satisfy the criteria of forming hairpin miRNAs, and that the mature miRNAs are present in one arm of the hairpin precursors; 3) The mature miRNA strand and its complementary strand (miRNA*) should contain 2-nt 3′ overhangs; 4) Hairpin precursors do not contain large internal loops or bulges; 5) Secondary structures of hairpins have free energy of hybridization ≤−18 kcal/mol; and 6) The number of mature miRNAs with predicted hairpins should be ≥5 in the alignment result [66, 67]. The expression of novel miRNA is produced by summing the count of those miRNAs with no more than three mismatches on the end of 5′ and 3′ and with no mismatch in the middle from the alignment result [66, 67]. We summarized the data analysis process in Additional file 2: Figure S1.

### Differential expression analysis of miRNAs

Firstly, the clear read numbers were normalized for the two samples (control and treatment) to get the expression of transcripts per million (TPM) of each miRNA [68] as follows:

$$ \mathrm{Transcripts}\ \mathrm{per}\ \mathrm{million}=\left(\mathrm{Actual}\ \mathrm{miRNA}\ \mathrm{count}/\mathrm{Total}\ \mathrm{count}\ \mathrm{of}\ \mathrm{clean}\ \mathrm{reads}\right)*1000000 $$

Differential expression of miRNAs was analyzed based on the sequence TPMs of the ABA treatment and control libraries. The fold-change was calculated by dividing the miRNA TPM in the ABA treatment library (A1D) by the miRNA TPM in the control library (C1D). *P*-value was calculated as described by Audic [69]. If the standard expression of a given miRNA is zero, its expression value was modified to 0.01. Sequences that had TPM below two in both samples were removed to reduce noise. Changes in the expression level of at least |log_2_fold-change (log_2_FC)| ≥0.25 were recognized as a response to ABA treatment, and significant difference in miRNA expression was assigned to sequences with *P*-value <0.05 and | log_2_FC| ≥1. miRNAs with |log_2_FC| <0.25 were considered to have no obvious change in expression levels. The statistic is calculated according the following equation:

$$ \mathrm{p}\left(\mathrm{y}\left|\mathrm{x}\right.\right)={\left(\frac{N_2}{N_1}\right)}^y\frac{\left(x+y\right)!}{x!y!{\left(1+\frac{N_2}{N_1}\right)}^{\left(x+y+1\right)}} $$

Where x indicates the number of reads across a miRNA in C1D, y indicates the number of reads across the corresponding miRNA in A1D, and N1 and N2 represent the total numbers of clean reads in C1D and A1D, respectively. Here, the *P* value indicates the probability of obtaining y counts in A1D given x counts in C1D.

### miRNA target prediction

To analyze the functions of the identified miRNAs, we searched for their target genes. To this end, miRNAs were mapped to the tomato genome database (ftp://ftpmips.helmholtzmuenchen.de/plants/tomato/tomato_genome/ITAG_annotation/ITAG2.3_release/ITAG2.3_cdna.fasta) [9], using criteria established in Allen et al. [34]. And the advances in miRNA target prediction has been improved [70]. Here, the targets for miRNAs were predicted using software Mireap 20.0. GO enrichment analysis of target gene candidates was carried out using the GO terms in the database (http://www.geneontology.org/).

### Stem-loop quantitative real-time PCR

Stem-loop quantitative real-time reverse transcription polymerase chain reaction (qRT-PCR) with SYBR Green was performed to verify the expression patterns revealed by miRNA-seq [71]. Thirty miRNAs were chosen for qRT-PCR, including the novel miRNAs identified in this study. Total RNA was isolated from leaves from control and ABA-treated plants, and first strand cDNA was synthesized using specific stem-loop primers listed in Additional file 1: Table S9. Each upstream primer contained 16–20 bases at 5′-end matches to appropriate miRNA, and each downstream reverse transcript primer contained 33–35 bases, comprising a 28 base stem loop  in common and 5–7 base matches to corresponding miRNA at 3′-end. The downstream reverse transcript primers contained the universal primer sequence CAGTGATGTTGCGGTCT.

0.1–1 μg of total RNA and 0.1 μg (1 μL) of the miRNA specific reverse transcription primer were used for reverse-transcription with the RevertAid First Strand cDNA Synthesis kit (RevertAid, Thermo scientific) following the manufacturer’s protocol. qRT-PCR was performed using SYBR® Green Realtime PCR Master Mix (Tiangen Biotech (Beijing) CO., LTD). The qRT-PCR reactions were carried out in the real-time PCR machine CFX96 (Bio-Rad). The reaction conditions were 95 °C for 2 min, followed by 45 cycle of 95 °C for 15 s, 45 ~ 50 °C (according to the Tm of each pair of primers) for 40 s, 60 °C for 30 s, and then fluorescence levels were measured at 60 °C. Three replicates were performed for each reaction. Relative expression level of a miRNA was measured in terms of threshold cycle value (Ct) and the small nuclear (snRNA) U6 RNA from *S. lycopersicum* was used as the internal control. The Ct value was normalized to U2 snRNA, in which ΔCt = Ct_miRNA_–Ct_U6_. The miRNA sequences, primer sequences were listed in Additional file 1: Table S6.

## Additional files

Additional file 1: Table S1. List of Known miRNAs identified in this study, and their expression levels and the predicted target genes. **Table S2.** List of novel miRNAs identified in this study, and their expression levels and the predicted target genes. **Table S3.** The list of novel miRNAs identified in this study that have been updated in miRNase 21.0 database. **Table S4.** The mature and precursor sequences of the novel miRNAs, and the corresponding secondary structures. **Table S5.** The differential expression levels in the control (C1D) and ABA-treatment (A1D) libraries of known miRNAs. **Table S6.** The differential expression levels in the control (C1D) and ABA-treatment (A1D) libraries of the novel miRNAs. **Table S7.** Selected miRNAs (TPM >10) and the expression levels of their targets (FPKM). **Table S8.** List of identified miRNAs that are linked to transcript factors and disease resistance genes. **Table S9.** Sequences of primers used for stem-loop qRT-PCR analysis. **Table S10.** Comparison of expression levels between RNA-seq expression and quantitative real-time PCR. (XLSX 482 kb) Additional file 2: Figure S1. Diagram of the data analysis process. (PDF 14 kb)

## Abbreviations

ABA: abscisic acid
A1D: ABA treatment after 1 day
C1D: water treatment after 1 day
AP2/EREBP: apetala 2/ethylene-responsive element-binding factor
bHLH: basic helix-loop-helix
bZIP: basic leucine zipper
LRR: leucine-rich repeat
MAP: mitogen-activated protein
MYB: myb proto-oncogene protein
NAC: no apical meristem/ATAF/CUP-shaped cotyledons
NBS: nucleotide binding site
RLK: receptor-like kinase
STK: serine/threonine-protein kinase
TFs: transcription factors
TPM: transcripts per millions
