# Supplementary figures and images for: Genomic profiling of exogenous abscisic acid-responsive microRNAs in tomato (Solanum lycopersicum)
Source: BMC Genomics. 2016 Jun 3;17:423. doi: 10.1186/s12864-016-2591-8 (PMC4891822; doi:10.1186/s12864-016-2591-8)

## Data analysis process

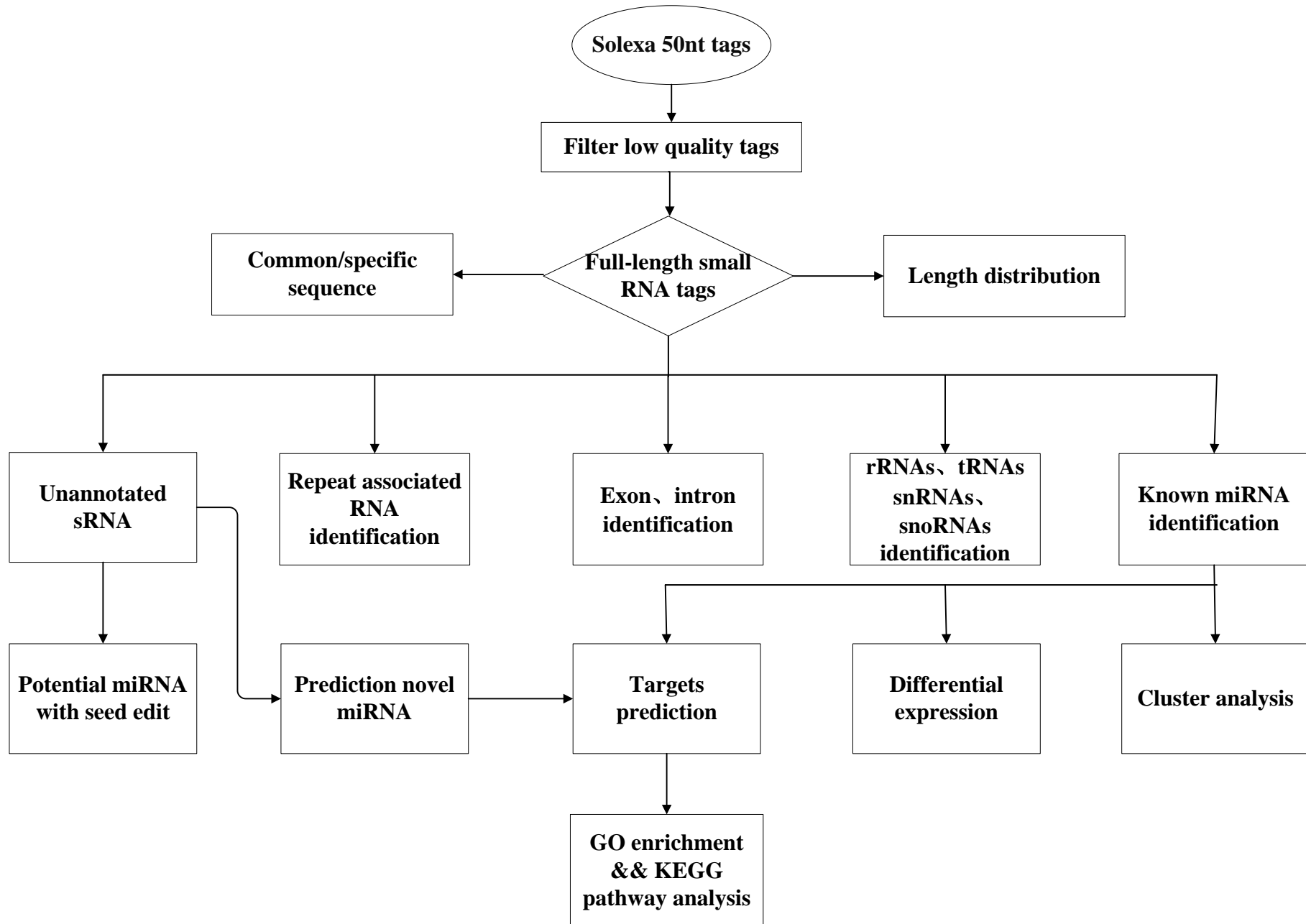

Supplement: Additional file 2: Figure S1. — Diagram of the data analysis process. (PDF 14 kb) [file 12864_2016_2591_MOESM2_ESM.pdf]
